# When Trabeculations Disrupt Conduction: Left Ventricular Noncompaction Presenting With Symptomatic Bradycardia and Isorhythmic Dissociation

**DOI:** 10.1002/ccr3.72825

**Published:** 2026-05-29

**Authors:** Talha Shamshad, Ryan Salama, Jurgen Shtembari, Mounica Vorla, Muhammad Asif, Sanchit Duhan, Mohammad Al‐Akchar, Osama Alsara

**Affiliations:** ^1^ Carle Foundation Hospital Urbana Illinois USA; ^2^ University of Minnesota Minneapolis Minnesota USA

**Keywords:** AV dissociation, bradycardia, cardiac MRI, cardiomyopathy, conduction system disease, left ventricular noncompaction

## Abstract

In young patients with unexplained symptomatic bradycardia, LVNC should be considered even when initial echocardiography is equivocal. Precise ECG interpretation is essential—AV dissociation driven by a ventricular escape rhythm must be distinguished from true AV block, as this distinction directly determines whether cardiac MRI, genetic evaluation, or device therapy is warranted.

## Learning Points

- Left ventricular noncompaction cardiomyopathy should be considered in young patients presenting with unexplained symptomatic bradycardia or unexplained conduction abnormalities.
- Precise ECG interpretation is critical: Isorhythmic dissociation due to a ventricular escape rhythm must be distinguished from true AV block, as the two carry different management implications including the decision to implant a permanent pacemaker.
- Cardiac MRI is critical for definitive diagnosis when echocardiographic findings are equivocal.
- Management of LVNC focuses on the management of associated clinical manifestations rather than on myocardial morphology alone.
- Careful rhythm surveillance and individualized risk stratification for sudden cardiac death are essential components of follow‐up.

## 1 Introduction

Left ventricular noncompaction cardiomyopathy (LVNC) is classified as a distinct cardiomyopathy by the European Society of Cardiology and is characterized by prominent myocardial trabeculations and deep intertrabecular recesses that communicate with the ventricular cavity. The prevailing hypothesis attributes LVNC to the arrest of normal myocardial compaction during early embryogenesis, although adaptive and acquired mechanisms have also been proposed. Genetically, LVNC is highly heterogeneous, with pathogenic variants most frequently involving sarcomeric and cytoskeletal genes such as MYH7, TTN, MIB1, and TAZ [1, 2].

The reported prevalence of LVNC varies widely, from < 0.1% in population‐based echocardiographic studies to over 10% in selected cohorts, reflecting differences in diagnostic criteria, imaging modalities, and referral bias [3]. Clinically, LVNC demonstrates marked phenotypic variability. Patients may remain asymptomatic or present with heart failure, ventricular and supraventricular arrhythmias, thromboembolic events, or sudden cardiac death [4]. Although arrhythmias are common, significant conduction system disease remains a relatively rare and incompletely characterized presentation [5].

We present a case of LVNC in a young adult with symptomatic bradycardia and isorhythmic dissociation, highlighting diagnostic challenges, the central role of CMR, and management considerations relevant to contemporary clinical practice.

## 2 Case History/Examination

A young woman in her 30s presented to the emergency department with acute onset dizziness, lightheadedness, and nausea. Her medical history was notable for post‐traumatic stress disorder. She denied prior cardiovascular disease, chest pain, dyspnea, or palpitations, although she recalled remote syncopal episodes during adolescence. There was no known family history of cardiomyopathy or sudden cardiac death.

On presentation, vital signs demonstrated bradycardia. A 12‐lead EKG revealed bradycardia with ventricular escape rhythm displaying left bundle branch block morphology, likely right ventricular in origin, producing AV dissociation with intermittent fusion beats (atria and ventricles beating at nearly identical rates, with hybrid beats occurring when a conducted sinus impulse coincides with the escape beat), without evidence of true atrioventricular block (Figure 1). Heart rate declined progressively from approximately 63 beats per minute on arrival to the low 40s during inpatient monitoring.

**FIGURE 1:**
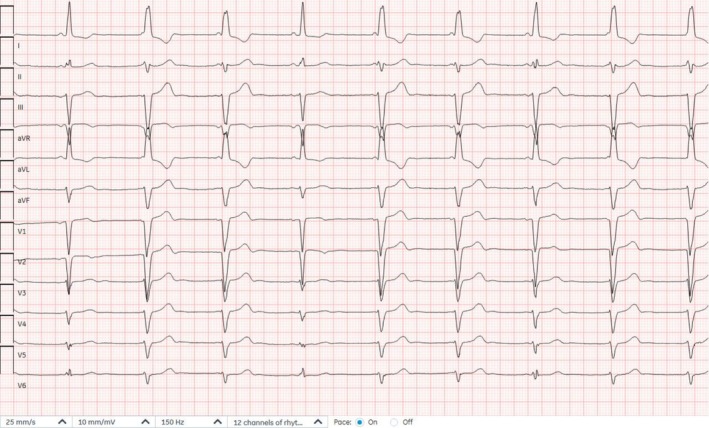
ECG showing junctional escape complexes displaying left bundle branch block morphology and isorhythmic dissociation.

## 3 Differential Diagnosis, Investigations and Treatment

Initial laboratory evaluation, including serial high sensitivity troponin, was negative, effectively excluding acute coronary syndrome or myocarditis. Lyme serology and autoimmune screening were negative. Mild anemia and electrolyte abnormalities were identified and corrected with IV fluids and potassium supplementation. Symptoms resolved with supportive management.

Transthoracic echocardiography demonstrated preserved left ventricular systolic function (ejection fraction 55%–60%), mild left atrial enlargement, mild mitral regurgitation, and suspected apical trabeculations (Figure 2). Given persistent isorhythmic dissociation and equivocal echocardiographic findings, further evaluation was pursued. Exercise stress testing demonstrated average functional capacity (10 metabolic equivalents) but was terminated early because of dizziness and fatigue, consistent with chronotropic incompetence (defined as failure to achieve 80% of the age‐predicted maximum heart rate despite adequate effort).

**FIGURE 2:**
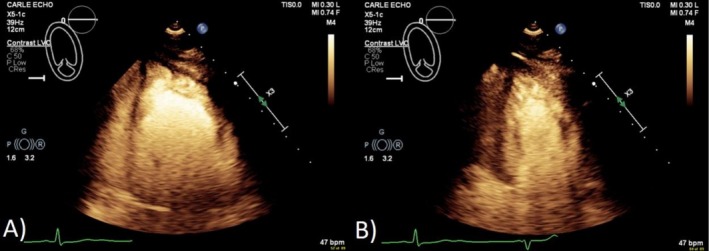
Echocardiographic apical two‐chamber view. (A) End‐diastolic view, (B) end‐systolic view showing trabeculations and intertrabecular recesses.

Coronary computed tomography angiography revealed a dilated left ventricle with prominent trabeculation and no evidence of coronary artery disease. Subsequent cardiac magnetic resonance imaging confirmed LVNC, demonstrating a noncompacted‐to‐compacted myocardial ratio of 4.5:1 in end‐diastole by the Petersen criteria (threshold > 2.3), left ventricular dilation, myocardial thinning, and mildly reduced systolic function (LVEF at 45%). No late gadolinium enhancement was identified (Figure 3).

**FIGURE 3:**
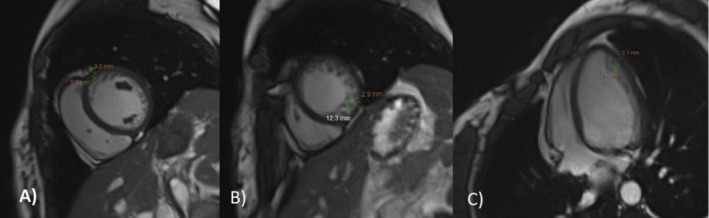
End‐diastolic cardiac magnetic resonance images showing noncompacted to compacted myocardial thickness ratio of higher than 2.3 meeting the Petersen criteria. (A) Short‐axis view at the level of papillary muscles, (B) short‐axis view at the level of mid‐ventricle, and (C) long‐axis view.

The patient was initiated on guideline‐directed medical therapy with an angiotensin receptor blocker. Beta‐blocker therapy was deferred because of symptomatic bradycardia, chronotropic incompetence, and borderline blood pressure. Plans were made to initiate a mineralocorticoid receptor antagonist and a SGLT‐2 inhibitor in the outpatient setting in accordance with contemporary heart failure guidelines [6]. Permanent pacemaker implantation was not pursued, as no true AV block was documented on monitoring and the bradycardia was attributed to a junctional escape mechanism rather than to primary conduction system failure. A 14‐day Holter monitor demonstrated predominantly normal sinus rhythm with one asymptomatic episode of non‐sustained ventricular tachycardia lasting 16 s. She was referred to electrophysiology consultation and genetic evaluation. Her symptoms improved over the following 2 months, and a repeat EKG at that time demonstrated normal sinus rhythm.

## 4 Conclusion and Results

This case highlights an uncommon presentation of LVNC with symptomatic bradycardia and isorhythmic dissociation in a young adult. It emphasizes the importance of comprehensive evaluation and multimodality imaging in patients with unexplained conduction disease. Early recognition allows for appropriate surveillance, genetic counseling, and individualized management aimed at reducing long‐term morbidity and mortality.

## 5 Discussion

LVNC is a diagnostically challenging cardiomyopathy with substantial phenotypic overlap with other myocardial disorders. While arrhythmias are frequently reported, conduction system disease is uncommon and largely described in isolated case reports and small series [5, 7]. The underlying mechanisms remain incompletely understood but may include myocardial disarray, fibrosis, or shared genetic substrates affecting both myocardial development and the conduction system [8].

Electrocardiographic abnormalities in LVNC are common but nonspecific. Left bundle branch block, repolarization abnormalities, and increased QRS voltages have been most frequently described [9]. In this case, the combination of symptomatic bradycardia with ventricular escape rhythm and isorhythmic dissociation displaying left bundle branch block in a young patient without structural heart disease and autoimmune phenomenon prompted further evaluation with advanced imaging. It is important to distinguish isorhythmic dissociation caused by a ventricular escape rhythm from true AV block. In this patient, sinus bradycardia allowed a subsidiary ventricular pacemaker, likely situated in the right ventricular Purkinje system given the LBBB morphology, to emerge as a protective response and usurp ventricular activation. The occurrence of fusion beats, produced when a conducted sinus impulse coincides with a ventricular escape beat, confirmed that antegrade AV conduction remained intact, whereas in true high‐grade AV block, the atrial rate exceeds the ventricular rate due to failed atrioventricular conduction. This distinction carries significant therapeutic implications, as true AV block typically mandates permanent pacing, while escape‐driven isorhythmic dissociation may not. In this patient, no AV block was confirmed on 14‐day Holter monitoring, and spontaneous resolution of the rhythm abnormality over 2 months supports a reversible or rate‐dependent conduction mechanism.

Conduction system involvement in LVNC has been reported in only a small number of cases. Patra et al. described biventricular noncompaction presenting with symptomatic complete heart block requiring permanent pacemaker implantation [5], and Yildiz et al. similarly reported complete AV block in biventricular noncompaction [7]. The present case is notable in that no true AV block was documented; the bradycardia arose from a ventricular escape mechanism and resolved spontaneously, emphasizing the importance of precise electrocardiographic characterization before committing to device therapy.

Transthoracic echocardiography remains the first‐line diagnostic modality for LVNC; however, its sensitivity is limited, particularly in visualizing the left ventricular apex. Cardiac MRI offers superior spatial resolution, improved apical visualization, and tissue characterization. It is increasingly regarded as the reference standard [10]. The Petersen criterion (a noncompacted‐to‐compacted myocardial ratio > 2.3 in end‐diastole) was clearly met in this patient supporting the diagnosis [11].

Risk stratification for sudden cardiac death in this patient incorporated several key factors. The presence of non‐sustained ventricular tachycardia on Holter monitoring and a mildly reduced LVEF of 45% placed her at intermediate risk. However, the absence of late gadolinium enhancement on CMR was reassuring, as myocardial fibrosis detected by LGE is an established independent predictor of adverse outcomes in LVNC [12]. Current guidelines for primary prevention ICD implantation generally require an LVEF ≤ 35% or other high‐risk features; given this patient's profile, device therapy was deferred in favor of electrophysiology consultation for individualized risk assessment and longitudinal surveillance.

Management of LVNC is directed toward clinical manifestations rather than toward the morphologic diagnosis itself. Heart failure therapy should follow contemporary GDMT. Anticoagulation should be considered on an individual basis, particularly in the presence of atrial fibrillation, prior thromboembolism, or ventricular thrombus [13]. Risk stratification for sudden cardiac death remains challenging; reduced ejection fraction, ventricular arrhythmias, and myocardial fibrosis on CMR are important considerations [12]. Device therapy should follow established indications for pacemaker or implantable cardioverter defibrillator implantation [6].

Genetic evaluation was recommended given the confirmed LVNC diagnosis in a young patient without an identifiable acquired cause. Pathogenic variants in genes such as MYH7, TTN, TAZ, and MIB1 have been associated with LVNC and may carry prognostic implications. Identification of a causative variant would facilitate cascade screening of first‐degree relatives, who may harbor subclinical disease and benefit from early surveillance and intervention [1, 2].

## Author Contributions

**Talha Shamshad:** writing – review and editing. **Ryan Salama:** writing – original draft. **Jurgen Shtembari:** project administration, writing – review and editing. **Mounica Vorla:** writing – review and editing. **Muhammad Asif:** validation, visualization. **Sanchit Duhan:** writing – review and editing. **Mohammad Al‐Akchar:** conceptualization. **Osama Alsara:** supervision.

## Funding

The authors have nothing to report.

## Ethics Statement

Ethics approval was not required for this single‐patient case report in accordance with institutional policy. All procedures were performed in accordance with relevant guidelines and regulations.

## Consent

Written informed consent was obtained from the patient for publication of this case report and accompanying clinical images.

## Conflicts of Interest

The authors declare no conflicts of interest.

## Data Availability

Data sharing not applicable to this article as no datasets were generated or analysed during the current study.
